# Psychometric Properties of the Chinese Version of the Fear of Negative Evaluation Scale-Brief (BFNE) and the BFNE-Straightforward for Middle School Students

**DOI:** 10.1371/journal.pone.0115948

**Published:** 2015-03-23

**Authors:** Jia Wei, Chunyu Zhang, Yadan Li, Song Xue, Jinfu Zhang

**Affiliations:** Faculty of Psychology, Southwest University of China, Chongqing, China; University of New South Wales, AUSTRALIA

## Abstract

**Background:**

The 12-item brief version of the Fear of Negative Evaluation Scale (BFNE) is one of the most widely used instruments to assess fear of negative evaluation. Recent evidence strongly supports the version composed of 8 straightforward items (BFNE-S), which possessesstronger psychometric properties. The purpose of the current study is to examine the psychometric prop-erties of the Chinese versions of the BFNE and BFNE-S for middle school students.

**Methodology:**

A total of 1009 middle school students were recruited in this study. The BFNE, the BFNE-S, the Friedman-Bendas Text Anxiety Scale (FBTAS), and the Social Anxiety Scale (SAS) were administered to 497 participants, and 52 participants were re-tested after four weeks. The BFNE, the BFNE-S, the Rosenberg Self-Esteem Scale (RSES), and the Balanced Inventory of Desirable Responding (BIDR) wereadministered to 492 participants. The BFNE and BFNE-S significantly cor-related with all the scales, supporting their convergent, divergent and concurrent validity.

**Principal Findings:**

The Cronbach's alpha of the BFNE (BFNE-S) was 0.864 (0.867) with 497 par-ticipants and 0.886 (0.844) with 492 participants, and the test-retest reliability coefficient was 0.791 (0.855) (ICC). Although the EFA identified a two-factor solution in which the 8 straightfor-ward items loaded on one factor and the 4 reversed items loaded on the other, the CFA, using a random intercept model to control the wording effect, supported a unidimensional factor struc-ture of the BFNE. Both EFA and CFA supported the unidimensional assumption of the BFNE-S. The correlations of the BFNE and BFNE-S were 0.929 and 0.952 in two samples.

**Conclusions:**

The Chinese versions of the BFNE and BFNE-S demonstrate adequate psychometric properties for assessing fear of negative evaluation. The results support their use among the Chinese middle school students. Considering its greater parsimony and excellent reliability and validity, the BFNE-S is a better tool.

## Introduction

Fear of negative evaluation was originally defined by Waston and Friend as “apprehension about other’s evaluations, distress over their negative evaluations, avoidance of evaluative situations, and the expectation that others would evaluate oneself negatively” [1]. This construct has been thought to be central to social anxiety disorder (SAD) [2]. An acknowledged hypothesis is that when SAD patients enter a social situation, they are inclined to negatively appraise their anxious symptoms (such as a racing heart and blushing) as evidence of poor social performance and to fear that these symptoms will be noticed and judged negatively by others [3]. Indeed, the fear of negative evaluation has been found to be significantly associated with measures of anxiety and distress among individuals with SAD [4]. Additionally, it has been associated with other psychological traits, such as low self-esteem [5]. Therefore, the assessment of the level of an individual’s fear of negative evaluation from others has clinical and non-clinical value.

Waston and Friend first developed a 30-item true-false scale, the Fear of Negative Evaluation Scale (FNE), to assess individuals’ trait levels of fear of negative evaluation [1]. Because the utility of the FNE is sometimes limited by its length, a brief version of the FNE (BFNE) containing 12 items selected from the original FNE was developed by Leary. Additionally, the response format of the BFNE was changed from the original true-false format to a 5-point Likert-type scale [6]. The psychometric properties of the BFNE were identical to the FNE; correlation between the total score of the BFNE and the score of the FNE was very high (r = 0.96, *p*<0.001). The Cronbach’s alpha coefficient of the BFNE was 0.90, and the 4-week test-retest reliability coefficient was 0.75 (Pearson’s correlation coefficient between the two measurement waves) [6]. The BFNE has therefore become a commonly used instrument to measure fear of negative evaluation because of its parsimony [7].

The measurement of fear of negative evaluation also has other practical applications, such as controlling common method bias (CMB, the bias due to the use of the same method [8]) Most researchers agree that CMB, which could potentially threaten the validity of conclusions about observed relationships, should not be ignored and should be controlled if possible [8]. Confessing to fear of negative evaluation is considered an undesirable disposition and has been shown to reversely correlate with social desirability [1], which is well known as a major source of CMB [9]. DiStefano and Motl demonstrated in their survey data that people who had higher levels of fear of negative evaluation suffered less social desirability [10]. Therefore, using the BFNE score as an alternative control variable could eliminate, at least partly, the influence of social desirability in survey data.

Interestingly, the scores of the BFNE may suffer from CMB as well. Because the scale is composed of both straightforward (positively worded) and reversed (negatively worded) items, extra covariance would be generated between items worded in the same way [11]. Consequently, although the theoretical construct of the BFNE is unidimensional, the two different factors, in which all straightforward items are loaded on one factor and all reversed items are loaded on the other, are always extracted in exploratory factor analysis (EFA).Additionally, the one-factor model in which all 12 items are loaded on one latent variable always fits poorly in confirmatory factor analysis (CFA) [8]. Previous studies have demonstrated that an incorrect or inadequate response to the reversed items might be caused by these situations [11].DiStefano and Motl’s study mentioned above only analyzed the 8 straightforward items of the BFNE instead of the full 12 items to “alleviate any possible confounding of the predictor scales with the method effect factor” [10]. Recently, Carleton and his colleagues systematically showed that the rationality of the 4 reverse-worded items of the BFNE should not be included in the measure or the analyses of the psychometric properties of the 8 straightforward-item version of the BFNE, called the BFNE-S. The results showed that the BFNE-S not only had excellent psychometric properties but also prevented the validity problems of the BFNE [7, 12].

Adolescence is an important stage in one’s physical, psychological and social development. Because of the increase in the frequency and importance of peer interactions, adolescents are vulnerable to SAD and fear evaluation [13]. Empirical studies in various cultural contexts have shown that the correlation coefficients between the scores of the BFNE and SAD assessed by different instruments are always high [14–16]. Furthermore, the questionnaire data collected from adolescents, compared with adults, are particularly vulnerable to CMB [17].Both the BFNE and the BFNE-S (especially the latter) might be valid and parsimonious instruments to help researchers control CMB in the adolescent population.

Although previous studies have mostly confirmed the adequate psychometric properties of the BFNE and BFNE-S, it is important to note that these psychometric properties should be tested in other populations (other than undergraduate/college student samples) and in different cultural contexts. However, according to our knowledge, no study has explored the psychometric properties of the BFNE or the BFNE-S in Chinese middle school students. Therefore, the purpose of the present study was to translate and validate the BFNE and BFNE-S in an adolescent Chinese-speaking sample and to assess the following psychometric properties: internal consistency, test-retest reliability, construct validity, and criterion validity.

## Methods

### Samples

This study was conducted in four phases, with different samples selected by convenience sampling methods (Table 1). The first phase included 20 subjects (sample 1) and was a pilot test to determine the semantic understanding of the instructions and the item formulation of the Chinese version of the BFNE (and the BFNE-S, incidentally). The second phase selected 497 subjects (sample 2) and aimed to explore the internal consistency, perform EFA and determine the convergent and divergent validity and criterion validity (the correlation between the BFNE and BFNE-S to the Friedman-Bendas Text Anxiety Scale and the Social Anxiety Scale). The third phase involved the participation of 492 subjects (sample 3) and aimed to assess internal consistency, conduct CFA and establish the criterion validity (the correlation between the BFNE and BFNE-S to the Rosenberg Self-Esteem Scale and the Balanced Inventory of Desirable Responding). The last phase selected 52 subjects (sample 4) from sample 2 after 4 weeks and aimed to evaluate reliability with the test-retest technique.

**Table 1:** Demographics of the 4 samples.

Demographic	Sample1	Sample2	Sample3	Sample4
characteristics	(N = 20)	(N = 497)	(N = 492)	(N = 52)
Gender (*N (%)*)				
female	10 (*50*)	248 (*49*.*9*)	269 (*54*.*7*)	31 (*59*.*6*)
male	10 (*50*)	245 (*49*.*3*)	223 (*45*.*3*)	21 (*40*.*4*)
missing		4 (*0*.*8*)		
Grade (*N (%)*)				
7	20 (*100*)	86 (*17*.*3*)	103 (*20*.*9*)	
8		90 (*18*.*1*)	88 (*17*.*9*)	
9		87 (*17*.*5*)	51 (*10*.*4*)	
10		92 (*18*.*5*)	105 (*21*.*3*)	52 (*100*)
11		80 (*16*.*1*)	90 (*18*.*3*)	
12		62 (*12*.*5*)	55 (*11*.*2*)	

#### Ethics Statement

The study was approved by the Institutional Review Board of the Southwest University of China. Written informed consent forms from the participants’ caretakers were requested before the surveys were completed. The participants were then given another version of the written informed consent after being fully informed of the research procedure to ensure that all participation was voluntary.

#### Materials

**Chinese Versions of Fear of Negative Evaluation Scale-Brief (BFNE) and the Straightforward-Item Version (BFNE-S)**: The original BFNE measures anxiety associated with perceived negative evaluation. This scale is composed of 12 items, and 4 items are reverse-worded items. Participants are asked to indicate the extent to which each item describes them on a 5-point Likert scale ranging from 1 (not at all) to 5 (extremely). Scores range from 12 to 60, with higher composite scores indicating greater fear of negative evaluation by others [6]. The BFNE-S is composed of 8 straightforward items selected from the BFNE with the same scale format. The scores range from 8 to 40, with higher composite scores indicating greater fear of negative evaluation by others [7]. For convenience, the item order and number of the BFNE-S were the same as the BFNE; the 8 items were item1, 3, 5, 6, 8, 9, 11, and 12 of the BFNE. The factor structure of the BFNE is uncertain; some researchers have found a unitary factor structure, whereas others have found a two-factor structure with factors characterized by straightforward and reverse-worded items [18]. In contrast, previous research has indicated that the factor structure of the BFNE-S is one-dimensional without controversy [7, 11, 12].

The “forward-backward” procedure was applied to the translation of the BFNE (and the BFNE-S, incidentally, as below in this section) from English to Chinese. Two native Chinese graduate students of psychology translated the questionnaire to Chinese independently, and two professional translators who were not familiar with the BFNE back-translated the scale to English. Then, the provisional version of the Chinese BFNE was reviewed by a panel of experts (including the student advisor and members of the research team). After the pilot test, the final Chinese versions of both the BFNE and BFNE-S were completed.

**Friedman-Bendas Text Anxiety Scale (FBTAS)**: The FBTAS is composed of 23 items and includes 3 subscales with 5 reversed-score items: the Tenseness subscale (TS, 6 items), the Social Derogation subscale (SDS, 8 items) and the Cognitive Obstruction subscale (COS, 9 items). Participants are asked to indicate the extent to which each item describes them on a 5-point Likert scale ranging from 1 (not at all) to 5 (extremely). Scores of the TS, SDS and COS range from 6 to 30, 8 to 40 and 9 to 45, respectively, with higher composite scores indicating greater test anxiety derived from physical complaints, social derogation (negative evaluation by peers, teachers and parents) and cognitive difficulties, respectively [19].The psychometric properties of the Chinese version of the FBTAS have been tested and widely used [20]. In the current study, the Cronbach’s alphas (95% CI, the same below) of the Chinese version of the TS, SDS and COS were 0.71 (0.67–0.75), 0.86 (0.84–0.88) and 0.81 (0.78–0.83), respectively.

**Social Anxiety Scale (SAS)**: The SAS is a subscale of the Multidimensional Anxiety Scale for Children (2^nd^edition) and includes 9 items using a 4-point Likert ranging from 0 (never) to 3 (often) to assess the presence of symptoms related to social anxiety disorders in youth aged 8 to 19 years. Scores range from 0 to 27 and higher scores indicate greater levels of social anxiety disorder [21]. In the current study, the Cronbach’s alpha of the Chinese version of the SAS was 0.81 (0.78–0.83).

**Rosenberg Self-Esteem Scale (RSES)**: The RSES is composed of 10 items. Participants are asked to indicate the extent to which each item describes them on a 4-point Likert scale ranging from 1 (not true of me) to 4 (very true of me). Scores range from 10 to 40, with higher composite scores indicating greater positive self-evaluation. The psychometric properties of the Chinese version of RSES have also been tested and widely used [22]. In this study, the Cronbach’s alpha of the RESE was 0.86 (0.84~0.88).

**Balanced Inventory of Desirable Responding (BIDR)**: The BIDR is a 40-item scale that uses a 7-point Likert scale ranging from 1 (not true) to 7 (very true) to assess the respondents’ tendencies to respond in a socially desirable manner. Scores range from 0 to 40 (only the extreme response scores 1, others score 0), and higher scores indicate greater levels of social desirability [23]. The psychometric properties of the Chinese version of the BIDR have been tested and widely used [24]. In the current study, the Cronbach’s alpha of the Chinese version of the BIDR was 0.84 (0.82–0.86).

### Analytic Strategy

First, the descriptive statistics (mean, standard deviation (SD), Skew and Kurtosis) of each item and the total scores of the BFNE, BFNE-S and all the criterion variables’ total scores were conducted with sample 2 and sample 3.The multivariate normal distribution test was conducted with the same samples as well. The internal consistency (assessed twice with samples 2 and 3) of the BFNE and BFNE-S was assessed using Cronbach’s alpha coefficient. Previous studies demonstrate that the two-way random intraclass coefficient for the absolute agreement of the single measure (ICC (A, 1)) could provide a more conservative estimate of the test-retest reliability than the Pearson correlation coefficient [25]. Therefore, the ICC (A, 1) was used in the present study to evaluate the test-retest reliability of the BFNE and BFNE-S in sample 4.

To analyze the construct of the BFNE and BFNE-S, EFA and CFA were performed. To meet the four recommendations proposed by Costello and Osborne [26], EFA was conducted on sample 2 and performed with maximum likelihood estimation with robust standard error (MLR) and geomin rotation [27]. The minimum average partial test (MAP) and the parallel analysis (PA) were used to determine the number of factors for retention [28]. The theoretical assumption of the MAP is the local independence among items in EFA. If the numbers of the principal components that were removed from the original correlation matrix of observed variables (items) are optimal, the average squared and 4th power of the partial correlation coefficients should be the smallest. This result would indicate that the removal of these principal components would make the observed variables reach optimal local independence. Therefore, the number of the removed principal components is the number of factors that should be retained [28]. There are two steps in PA. First, a set of random simulated data with the same variables and sample size as the raw data is generated. Second, the average eigenvalues of the random data are computed, and the eigenvalues of the raw data are compared with the average eigenvalues. If the number N eigenvalues of the raw data are smaller than the same number N average eigenvalues of the random data, this result indicates that the covariance due to this factor is smaller than the covariance due to the random factor, which might have no substantial significance. Therefore, the N-1 factor(s) should be retained to ensure that the factor(s) retained has (have) substantial significance [28] (this process is described in reference 28).

Based on the results of EFA, CFA was conducted primarily to test the most suitable model of the BFNE among 7 alternative models in sample 3. Model 1 (M1) was a single-factor model in accordance with the original unidimensional assumption. Model 2 (M2) was a 2-factor model identified by the previous EFA. Model 3 (M3) was a random intercept model [9]. In M3, all the items’ scores were influenced differently (with different factor loadings) by only one trait factor that represented the individual’s latent trait of fear of negative evaluation. Additionally, all the items were equally influenced (with the same factor loadings) by a random intercept factor to capture the systematic variance of the different participants in response to the straightforward and reverse-worded items. Therefore, the covariance between the items due to method effect derived from the straightforward and reversed wording could be separated from the covariance due to the latent trait. Model 4 (M4) and Model 5 (M5) were method effect models with a reference method in which the straightforward-worded method or the reverse-worded effect method was chosen as the reference method, respectively [29]. In M4 (M5), all the items’ scores were influenced differently by only one trait factor that represented the individual’s latent trait of fear of negative evaluation. Additionally, all the reversed (straightforward) items were equally influenced by another latent variable to capture the extra systematic variance due to the use of reversed (straightforward) method to measure the same latent trait as the straightforward (reversed) method. Model 6 (M6) and Model 7 (M7) were method effect models with common trait factors in which the straightforward-worded method or the reverse-worded effect was chosen as the reference method, respectively [30]. In M6 (M7), all the items’ scores were influenced differently by only one trait factor that represented the individual’s latent fear of negative evaluation. Additionally, all the reversed (straightforward) items were equally influenced (the factor loadings were set to 1) by another latent variable, which equally influenced all the straightforward (reversed) items. However, the factor loadings were set to −1 to capture the extra systematic variance due to the use of the reverse-worded (straightforward) method to measure the same latent trait as the mean effect of using both the straightforward and reverse-worded methods. M4 and M5, similar toM6 and M7, are equivalent models derived from the algebraic equivalence of the model parameters. In other words, the equivalent model will produce the same covariance, correlation and residual matrix if it fits the same data. Therefore, the χ^2^ values and the fit indices among the equivalent model are the same [31].Capitalizing on the same sample, the factor construct of the BFNE-S was also tested. Model 8 (M8) was a single-factor model in which all 8 straightforward items loaded on one latent variable that represented the fear level of negative evaluation. Convergent validity was assessed by correlations between the BFNE and BFNE-S and the SDS. Divergent validity was assessed by correlations between the BFNE and BFNE-S and the TS and COS. Both were conducted on sample 2. According to the Multitrait-Multimethod assumption proposed by Campbell and Fiske, the expected correlation coefficients of convergent validity should be significantly higher than the correlation coefficients of divergent validity [32].

Concurrent validity was assessed by correlations between the BFNE and BFNE-S and the SAS and RSES in sample 3. According to the previous studies, the expected correlation between the BFNE and BFNE-S and the SAS should be moderately or highly positive [14–16]but slightly negative with the RSES [10] and BIDR [1]. The correlation between the total scores of BFNE and BFNE-S was also conducted to provide more evidence of concurrent validity.

The descriptive statistics, Cronbach’s alpha, ICC (A, 1), MAP and all of the bivariate correlation analyses were performed with IBM SPSS Statistics 21.0 with pairwise deletion of the missing data (after deletion, 491 cases in sample 2 and 482 cases in sample 3 remained). The EFA, PA, CFA, multivariate normal distribution test and difference significance test of convergent and divergent validity were performed with Mplus 7.0.Because the missing data and the normality assumption were not sustained in the current study, the full-information maximum likelihood (FIML) estimator with robust standard error (MLR) and Bayesian estimator (used in CFA of M7 and M8) were employed with the undeleted sample 2 and sample 3 [27]. Five fit indices were assessed with the following criteria [33]: non-significant chi-square statistic (χ^2^), a cut-off value close to 0.90 for both the comparative fit index (CFI) and the Tucker–Lewis Incremental Fit Index (TLI), and a value lower than 0.08 for both the standardized root mean-square residual (SRMR) and the root mean-square error of approximation (RMSEA). The chi-square statistic is significant because it is highly sensitive to the sample size [34] and distribution of the data [35]. Therefore in this study, this result does not definitely determine the model’s fit.

## Results

### Descriptive Statistics

The mean, SD, skew, and kurtosis of each item, the total score of the BFNE and BFNE-S, and the results of the multivariate normal distribution test of samples 2 and 3 are shown in Table 2. The multivariate skew of the BFNE (BFNE-S) was 13.19 (3.05) and 11.65 (6.13) in sample 2 and sample3, respectively. The multivariate kurtosis of the BFNE (BFNE-S) was 207.89 (99.44) and 204.30 (98.47) in sample 2 and sample3, respectively. All the P-values of these coefficients were less than 0.001, indicating that the normality assumption of the current datasets was not sustained.

**Table 2:** The descriptive statistics of the items and total score of the BFNE and BFNE-S

	Sample 2 (n = 491)^b^				Sample 3 (n = 482)^b^			
	Mean	SD	Skew	Kurtosis	Mean	SD	Skew	Kurtosis
**I1**	3.20	1.22	0.10	−1.04	3.67	1.15	−0.71	−0.30
**I2** ^**a**^	4.08	1.06	−1.13	0.72	3.66	1.10	−0.71	−0.14
**I3**	2.81	1.22	0.19	−0.94	3.25	1.21	−0.56	−0.90
**I4**	4.24	0.98	−1.27	1.10	3.74	1.10	−0.69	−0.26
**I5**	3.05	1.31	−0.01	−1.15	3.46	1.26	−0.49	−0.80
**I6**	2.56	1.21	0.45	−0.67	3.06	1.22	−0.02	−0.99
**I7**	3.98	1.08	−0.97	0.34	3.41	1.20	−0.38	−0.84
**I8**	3.19	1.13	−0.05	−0.71	3.79	1.03	−0.78	0.09
**I9**	3.07	1.21	0.01	−0.98	3.63	1.13	−0.58	−0.44
**I10**	4.07	1.05	−1.24	1.20	3.51	1.12	−0.50	−0.47
**I11**	3.04	1.33	−0.01	−1.12	3.28	1.30	−0.22	−1.14
**I12**	3.10	1.30	−0.02	−1.08	3.76	1.10	−0.66	−0.32
**BFNE-S**	24.02	7.15	0.12	−0.70	27.90	6.50	−0.23	−0.36
**BFNE**	40.38	8.96	−0.15	−0.22	42.22	9.26	−0.25	−0.24

a: item 2,4,7,10 were reverse-coded

b: the two samples are different from the Table 1 as the missing data

### Reliability

In sample 2, the Cronbach’s alpha coefficient of the BFNE and BFNE-S were 0.864 (0.846–0.881) and 0.867 (0.849–0.884), respectively. In sample 3, the two coefficients were 0.886 (0.870–0.900) and 0.844 (0.822–0.864), respectively. In sample 4, the ICC (A, 1) between a 4-week test-retest was 0.791 with BFNE and 0.855 with BFNE-S.

### Exploratory factor analysis (EFA)

For the BFNE, both MAP and PA indicated that a minimum of 2 factors should be maintained (Table 3). With MAP, after the first two principal components were removed from the original correlation matrix, both the average squared and fourth power partial correlation coefficients were the smallest (0.0314 and 0.0036, respectively), indicating that no further components needed to be extracted from the matrix. With PA, the third eigenvalue of raw data was smaller than the average of the third average eigenvalue of the random data with the same number of variables and sample size (0.839 vs. 1.141).This finding indicated that the variance of the items due to the third common factor was smaller than the variance due to random factors, suggesting that only 2 factors should be extracted from the original data. For the BFNE-S, both MAP and PA indicated that a minimum of 1 factor should be maintained (Table 3) according to the following criteria: the smallest average squared and fourth power partial correlation were 0.0370 and 0.0021, respectively, and the second eigenvalue of raw data was 0.834, which was smaller than 1.121, the average of the second eigenvalues from the random data, with the same number of variables and sample size.

**Table 3:** The results of MAP and PA (partly)

RetainedComponent	BFNE				BFNE-S			
	MAP		PA		MAP		PA	
	SASPC	SAFPC	RE	AEFRA	SASPC	SAFPC	RE	AEFRA
None^a^	0.1275	0.0273			0.2103	0.0494		
1	0.0468	0.0047	4.845	1.260	**0.0370**	**0.0021**	**4.165**	**1.191**
2	**0.0314**	**0.0036**	**2.213**	**1.193**	0.0612	0.0097	0.834	1.121
3	0.0392	0.0037	0.839	1.141	0.0963	0.0457	0.758	1.066
……	……	……	……	……	……	……	……	……
6	0.0963	0.0487	0.552	1.012	0.4535	0.3820	0.442	0.928
7	0.1453	0.0895	0.527	0.975	1.0000	1.0000	0.374	0.881
8	0.2082	0.1216	0.431	0.937	NA	NA	0.315	0.821
……	……	……	……	……	NA	NA	NA	NA
11	1.0000	1.0000	0.305	0.815	NA	NA	NA	NA
12	NA	NA	0.239	0.762	NA	NA	NA	NA

SASPC = smallest average squared partial correlation, SAFPC = smallest average 4th power partial correlation, RE = raw eigenvalue, AEFRA = average of the eigenvalues from random data.

a: The values of MAP in “None” row indicating that no principal component was partial out from the original correlation matrix.

Because 1) the theoretical model of the BFNE and BFNE-S were unidimensional and 2) MAP and PA were recommended widely by statisticians as the upper bound of the number of remaining factors [28] and based on the recommendation of an anonymous reviewer, 1- to 3-factor models were tested with the BFNE and 1- and 2-factor models were tested with the BFNE-S. Although the 3-factor model of the BFNE and the 2-factor model of the BFNE-S had excellent fit indices, item 5 and item 12 in both models were cross-loading, which complicates the interpretations of the factor structure. Therefore, the 2-factor model of the BFNE and 1-factor model of the BFNE-S, which had second-best fit indices, were chosen as the optimal models (Table 4). The fit indices and factor loadings with geomin rotation are shown in Table 4. Although the 2-factor BFNE model was chosen as the optimal model, it was inconsistent with the theoretical model. The 2-factor solution of the EFA was consistent with many previous studies that have used English and other language versions of the BFNE and were found to be contaminated by CMB [14, 36].This finding suggests that the Chinese version of the BFNE might have suffered from CMB as well. Although the TLI and RMSEA were slightly poor, the other fit indices and the factor loadings were good in the 1-factor model of the BFNE-S, which was consistent with Carleton’s studies [7, 12].

**Table 4:** The EFA and CFA’s fit indices of the alternative models

Model		χ^2^	*df*	CFI	TLI	SRMR	RMSEA(90% CI)
BFNE	EFA 1-factor	813.113	54	0.609		0.130	
	EFA 2-factor	**182.408**	**43**	**0.928**		**0.038**	
	EFA 3-factor	118.778	33	0.956		0.039	
	CFA M1	312.000	54	0.827	0.788	0.066	0.099 (0.088–0.109)
	CFA M2	187.219	53	0.910	0.888	0.046	0.072 (0.061–0.083)
	CFA-M3 ^a^	**186.806**	**53**	**0.919**	**0.900**	**0.045**	**0.072 (0.061–0.083)**
	CFA M4-M7^b^	237.760	63	0.883	0.877	0.087	0.075 (0.065–0.085)
BFNE-S	EFA 1F	**113.326**	**20**	**0.909**		**0.048**	
	EFA 2F	55.282	13	0.959		0.031	
	CFA M8	108.839	20	0.898	0.857	0.047	0.095 (0.078–0.113)

a: According to the reference 9 the raw data (unreversed) was used to fit the M3.

b: According to the reference 31 the model fit indicators of M4-M7 were the same.

### Confirmatory factor analysis (CFA)

With regard to the BFNE, given that 1) the theoretical assumption of the structure was unidimensional; 2) the ratio between trait variance and the method effect variance (1: 0.076) was rational; and 3) the magnitude of the factor loading due to the trait factor was greater than the factor loading due to the method factor, M3 was chosen as the optimal model. With regard to the BFNE-S, because there was no other method compared with the straightforward-scored method, only M8 was tested. Except for the SRMR, the fit indices were slightly poor. After testing the model fit, the Bayesian estimator was conducted to calculate the credibility interval of the items’ factor loadings based on M3 and M8, respectively. The results of all the models’ fit indices are shown in Table 3, and the factor loadings are shown in Table 5. Note that the factor loadings with the RI factor (M3) presented in Table 5 were not equal due to the STDYX output by Mplus, which was thought to be the most appropriate standardized solution of the measurement model. With regard to the credibility interval, all credibility intervals overlapped, indicating that these factor loadings were not statistically different.

**Table 5:** the factor-loadings of EFA and CFA

items	EFA^a^			CFA^b^		
	full items		8 items	full items with M3		8 items with M1
	F1	F2	F1	T (95% CrI)^c^	RI (95% CrI)	T (95% CrI)^c^
**1**	**0.67**	-0.01	**0.68**	0.56(0.49–0.62)	0.24 (0.21–0.27)	0.54 (0.46–0.61)
**2**	−0.04	**0.66**		−0.66(−0.72–0.60)	0.25 (0.22–0.28)	
**3**	**0.66**	-0.08	**0.65**	0.60(0.54–0.66)	0.23 (0.20–0.26)	0.68 (0.62–0.73)
**4**	0.02	**0.78**		−0.73(−0.77–0.67)	0.25 (0.22–0.28)	
**5**	**0.71**	0.06	**0.68**	0.67(0.61–0.72)	0.22 (0.19–0.25)	0.68 (0.62–0.74)
**6**	**0.71**	**0.11**	**0.66**	0.58(0.52–0.64)	0.23 (0.20–0.26)	0.65 (0.59–0.71)
**7**	0.03	**0.88**		−0.71(−0.75–0.65)	0.23 (0.20–0.26)	
**8**	**0.72**	-0.05	**0.74**	0.55(0.48–0.61)	0.27 (0.23–0.30)	0.59 (0.52–0.65)
**9**	**0.73**	-0.05	**0.75**	0.69(0.64–0.74)	0.24 (0.21–0.28)	0.72 (0.66–0.77)
**10**	−0.01	**0.82**		−0.70(−0.74–0.64)	0.25 (0.22–0.28)	
**11**	**0.63**	0.01	**0.63**	0.62(0.55–0.68)	0.21 (0.19–0.24)	0.66 (0.59–0.71)
**12**	**0.60**	0.06	**0.57**	0.43(0.35–0.51)	0.25 (0.22–0.29)	0.51 (0.43–0.58)

a: boldface means significant at 5% level in EFA

b: all the factor loadings presented were significant at 5% level in CFA

c: CrI = Credibility Interval estimated by Bayesian estimator

### Convergent, Divergent and Concurrent Validity

All means and SDs of the criteria variables and the correlation coefficients between the total scores of the BFNE, the BFNE-S and these variables are shown in Table 6. Convergent and divergent validity pertain to construct validity, and concurrent validity pertains to criterion validity. The difference significance test showed that the correlation coefficients between the BFNE/BFNE-S and the SD were significantly higher than the correlation coefficients between the BFNE/BFNE-S and TS and COS. Pearson’s correlation coefficients of the relationship between the BFNE and the BFNE-S were 0.929 in sample 2 and 0.952 in sample 3(*p*s < 0.001).

**Table 6:** The results of the validity test

Criterion-Variable	n^a^	BFNE	BFNE-S	mean	SD
SDS	491	0.449***	0.468***	22.62	7.15
TS	491	0.284***	0.292***	16.35	4.63
COS	491	0.258***	0.266***	24.63	5.98
SAS	491	0.631***	0.610***	15.13	5.32
RSES	482	−0.160***	−0.131**	29.51	4377
BIDR	482	−0.102*	−0.103*	9.00	5.91

*: p < 0.05,

**p < 0.01

a: because of the missing data, the samples in this table are different from the Table 1

## Discussion

The current study provides the first comprehensive analysis of the psychometric properties of both the BFNE and the BFNE-S in which only 8 straightforward items of the BFNE were analyzed for Chinese middle school students. The results confirmed adequate psychometric properties of the BFNE and the BFNE-S after using the random intercept model to control CMB caused by the straightforward and reversed wording effect.

Regarding the reliability of the BFNE, the Cronbach’s alpha coefficients were 0.864 (0.846–0.881) and 0.886 (0.870–0.900) in sample 2 and sample 3, respectively. The results were consistent with findings from other studies using different language versions, in which the same coefficients ranged from 0.79 to 0.90 [14, 18, 35]. For the BFNE-S, the Cronbach’s alpha coefficients were 0.867 (0.849–0.884) and 0.844 (0.822–0.864) in sample 2 and sample 3, respectively, which were lower than previous studies, in which the same coefficients were 0.95 [7].The confidence interval of the Cronbach’s alpha coefficients of the BFNE and BFNE-S with the same sample overlapped, indicating that the two coefficients were not significantly different. Specifically, the internal consistency of the BFNE-S was as excellent as that of the BFNE, although 4 theoretical homogeneous items were excluded, which generally leads to lower internal consistency. This result implied that the 4 reversed items of the BFNE might have some problems, such as low homogeneity with the straightforward items. For the BFNE, the ICC of the 4-week test-retest reliability was 0.791, which was higher than the Iranian version (0.71) that used the same indicator [36] and the original version (0.75) that used the Pearson correlation coefficient [14]. For the BFNE-S, the same coefficient improved to 0.855, indicating that the straightforward items might be more stable than the reversed items. Specifically, using the BFNE-S to assess an individual’s level of fear of negative evaluation may have more stable results. Combined with the two reliability indices in the current study, the BFNE-S performed better than the BFNE.

With regard to factorial validity, two relatively strict strategies (MAP and PA) were employed to determine the number of factors that remained in EFA. For the BFNE, considering the unidimensional assumption, the results of the 2-factor solution suggested by the MAP and PA were inadequate. Therefore in the current study, it was reasonable to assume that neither the MAP nor the PA could exclude the common method bias caused by the straightforward and reversed wording effect of the raw data. This issue demonstrates that the structure of the BFNE might be distorted by CMB. To confirm this hypothesis, 7 alternative models were tested in CFA. Considering that the theoretical structure of the BFNE was unidimensional and the variance caused by the method effect should be much smaller than the variance caused by the trait (0.21:1 in M3), the random intercept model (M3) was chosen as the optimal model, although the 2-factor model (M2) identified by EFA fit slightly better than M3. The result of the Bayesian estimator showed that the credibility interval of all the items’ factor loadings in M3 were acceptable, which also indicates that the random intercept model was a rational model. With regard to the BFNE-S, although the original fit indices of both EFA and CFA (M8) were slightly poor, the indices could be significantly improved by allowing the correlation between the residual errors of item 8 and item 9. An anonymous reviewer argued that this treatment might cause theoretical changes to models.

In support of the convergent and divergent validity, the correlation coefficients between the total scores of the BFNE, BFNE-S and the SDS (convergent validity) were significantly higher than the correlation coefficients between the total scores of the BFNE/BFNE-S and the TS and COS (divergent validity) at the 0.1% significance level.

Previous studies have shown that the BFNE has concurrent validity with various instruments that measure social anxiety disorder [14–16]. In the present study, both the BFNE and BFNE-S were found to be significantly highly positively correlated with the SAS measure of social anxiety. This highly positive correlation coefficient (r = 0.631 and r = 0.610, respectively) indicates that the fear of negative evaluation is one of the core components of social anxiety disorder and can be regarded as an element of social anxiety. The correlations between both the BFNE and BFNE-S and the RSES and BIDR were slightly positive and negative, respectively, which was consistent with previous studies [1, 5]. The results confirm that the fear of negative evaluation is associated with low self-esteem and represents undesirability in society. It is interesting that fear of negative evaluation has also been shown to be a major motivation of impression management [37, 38], which is consistently positively correlated with social desirability. Logically, the correlation between the fear of negative evaluation and social desirability should be positive. Therefore, the relationship among these three traits should be investigated in subsequent studies.

Several limitations to the current study should be noted. First, with regard to the BFNE-S, the ICC interval was 0.707–0.923 compared with the BFNE’s interval (0.409–0.909), which indicated that the reversed-coded items had much more variance than the straightforward-coded items. Therefore, the ICC of the BFNE in the current study should be treated and explained discretely. The poor stability of the reverse-coded items further supports the removal of these items. Second, because the random intercept factor was an unmeasured factor in the BFNE, which indicated that the meaning of systematic variance was vague, the exact meaning of the random intercept factor should be explored in the future. With regard to the BFNE-S, given that 1) the phrase “others may be thinking about me” (item 8) and “the impression one makes” (item 9) are very similarly phrased in Chinese and 2) the proximity effect might produce additional covariance because these two items with the same scoring direction are next to each other in the questionnaire [39], there might be some undesirable covariance between these items. Whether changing the order of the items, especially segregating item 8 and item 9 from each other, could eliminate the undesirable covariance between these two items was not evaluated. This examination would be very useful in future studies. Third, the measurement invariance of the Chinese version and other language versions of the BFNE, BFNE-S and the Chinese version of the BFNE and the BFNE-S in other populations, such as adults, could not be examined because there were no reliable data from other populations or countries. Fourth, discriminant validity was not tested in this study. Fifth, the predictive validity of the BFNE and BFNE-S was not confirmed because of an unavoidable defect in cross-sectional studies. Future studies may examine these issues.

In conclusion, although the Chinese versions of the BFNE and BFNE-S are psychometrically supported, the latter 1) is composed of fewer items; 2) possesses better test-retest reliability; and 3) better controls unrelated measurement error. Additionally, there was a significant correlation between the BFNE and BFNE-S. Therefore, the BFNE-S appears to be a more valid and parsimonious instrument to measure fear of negative evaluation among middle school students in China.

## Supporting Information

S1 File Sample 2. Data for EFA, convergent, divergent and criterion validity (SAS). S**ample 3**. Data for CFA and criterion validity (RSES, BIDR). **Sample 4**. Data for test-retest reliability.
(RAR)
